# An inter-model assessment of the role of direct air capture in deep mitigation pathways

**DOI:** 10.1038/s41467-019-10842-5

**Published:** 2019-07-22

**Authors:** Giulia Realmonte, Laurent Drouet, Ajay Gambhir, James Glynn, Adam Hawkes, Alexandre C. Köberle, Massimo Tavoni

**Affiliations:** 1 RFF-CMCC European Institute on Economics and the Environment (EIEE), Centro Euro-Mediterraneo sui Cambiamenti Climatici, Milan, 20144 Italy; 2 Imperial College London, Grantham Institute, London, SW7 2AZ UK; 3 MaREI Centre, Environmental Research Institute, University College Cork, Cork, T23 XE10 Ireland; 4 Department of Management, Economics and Industrial Engineering, Politecnico di Milano, Milan, 20156 Italy

**Keywords:** Climate-change mitigation, Energy and society

## Abstract

The feasibility of large-scale biological CO_2_ removal to achieve stringent climate targets remains unclear. Direct Air Carbon Capture and Storage (DACCS) offers an alternative negative emissions technology (NET) option. Here we conduct the first inter-model comparison on the role of DACCS in 1.5 and 2 °C scenarios, under a variety of techno-economic assumptions. Deploying DACCS significantly reduces mitigation costs, and it complements rather than substitutes other NETs. The key factor limiting DACCS deployment is the rate at which it can be scaled up. Our scenarios’ average DACCS scale-up rates of 1.5 GtCO_2_/yr would require considerable sorbent production and up to 300 EJ/yr of energy input by 2100. The risk of assuming that DACCS can be deployed at scale, and finding it to be subsequently unavailable, leads to a global temperature overshoot of up to 0.8 °C. DACCS should therefore be developed and deployed alongside, rather than instead of, other mitigation options.

## Abstract (web summary)

Direct Air Carbon Capture and Storage (DACCS) is not considered in Integrated Assessment Models. Here the authors make comparisons using multi-model regarding the role of DACCS in 1.5 and 2 degree scenarios and find that DACCS allows to postpone mitigation and reduce the climate policy costs.

## Introduction

The Paris Agreement has set ambitious objectives to keep global warming well below 2 °C, and the scientific debate has recently focused on the pursuit of 1.5 °C targets^1^. Given the current level of CO_2_ emissions approaching 40 GtCO_2_/year^2^ and the delay of global mitigation efforts, large-scale removal of CO_2_ from the atmosphere will likely be needed. Studies on Negative Emission Technologies (NETs), also referred to as Carbon Dioxide Removal (CDR), have been conducted for almost two decades, but the topic has received more attention since the IPCC’s Fifth Assessment Report (AR5) published in 2013^3^.

Bioenergy with Carbon Capture and Storage (BECCS) and afforestation are considered the most likely options to realise negative emissions, and have been largely investigated in many Integrated Assessment Modeling (IAM) studies. However, concerns about the sustainability of biological strategies and competition with food, water use, and ecosystems^4,5^ have led to a focus on alternatives to sequester carbon from the atmosphere. Direct Air Carbon Capture and Storage (DACCS) is a complementary technology; it can capture the CO_2_ produced by distributed sources, is modular and does not have major water and land interactions^5–7^, while competing for geological storage with other carbon sequestration options. To date, only a few modeling exercises have included CDR measures other than afforestation and BECCS, and never in an inter-model comparison^8–10^.

DACCS captures CO_2_ from ambient air and subsequently stores it. Despite being at an early stage of development, it is gaining increasing attention, with multiple companies developing designs at a commercial scale^11–13^. Compared to other sequestration options, capturing CO_2_ directly from the air presents a number of advantages. Like other NETs, it can address distributed emissions, such as those from transport, aviation and intensive industrial sectors, together accounting for almost 50% of total emissions^14^. Designs for DACCS plants are diverse, some of them being modular, which extends a prospect of more rapid scaling^15^. Most demand little land, although some might still require significant although reduced water inputs^5,16^. Their main drawback is the low concentration of carbon dioxide in ambient air, meaning that a large amount of energy input is required.

Two groups of technologies can be identified as most promising, from a technical and economic perspective. The first (hereafter named as DAC1) is based on using water solutions containing hydroxide sorbents with a strong affinity for CO_2_, such as sodium hydroxide, calcium hydroxide and potassium hydroxide^6,17–20^. The second (DAC2) uses amine materials bonded to a porous solid support^12,21–23^. A wider range of solid sorbents are being investigated (e.g. ionic membranes^24^, zeolites^25^, solid oxides^26^), but were not included in our analysis as they are still at the research stage. Hydroxide solutions require high-temperature heat to be regenerated (*T* > 800 °C), which can be provided by burning natural gas, while amine adsorbents require only approximately 85–120 °C, meaning that waste heat can be used. While DAC1 is based on large scale plants, capturing 1 MtCO_2_/year, DAC2 has a modular design, that might be suitable for mass production and potentially rapid cost reduction (see Methods). DAC1 technology is more mature. It employs equipment already developed and adopted in other sectors (e.g.pulp and paper industry): the major expenditure is related to capital investment for building plant facilities, with limited potential for future cost reduction. Conversely, for DAC2 the potential for design and cost improvement is arguably higher, but issues regarding sorbent degradation may lead to high operational expenditure. Currently there is huge uncertainty on DACCS economics, with estimates in the literature ranging from 100 to 1000 $/tCO_2_^7^, according to the different designs proposed and the purity level of the captured CO_2_/year^27^.

This paper investigates the role of DACCS as part of a diversified portfolio of mitigation strategies, so as to inform policy-makers about its potential in low stabilization scenarios. We incorporate detailed technical and economic characteristics of a range of DACCS technologies into two IAMs, using the latest available estimates from the literature^17^. Considering the large degree of uncertainty around this new technology, besides parametric sensitivity analysis, we implement a model comparison adopting two complementary IAMs: a bottom-up technology rich model (TIAM-Grantham) and a hybrid, economy-climate model (WITCH). We also explore the requirements in terms of resource use and the consequences of DACCS failure on global temperature.

Results suggest that DACCS allows an easing of near term mitigation, and can significantly reduce climate policy costs. DACCS complements, rather than substitutes other negative emission technologies. The key factor governing the role of DACCS compared to other mitigation and negative emissions strategies is the rate at which DACCS capacity can be ramped up. Such a massive deployment requires a major refocusing of the manufacturing and chemical industries for sorbent production, and a large need for electricity and heat. Assuming that DACCS can be deployed at scale, and finding it to be subsequently unavailable, leads to a global temperature overshoot of up to 0.8 °C. Our results therefore show the considerable potential of DACCS but also highlight a large number of challenges which commend caution and further scrutiny.

## Results

### Modeling DACCS within integrated assessment models

Compared to previous studies including DACCS in IAMs^8–10,16^, we distinguish among different technology options, namely DAC1 and DAC2 as described above. We include the use of waste heat to operate the amine-based plants, recovering it from energy-intensive industries and renewable power plants. In order to define energy inputs and cost assumptions, we combine data from the available literature with estimates from the companies operating the first demonstration plants. Given the large uncertainty, we specify scenarios which have high and low levels of the key techno–economic parameters (see Table 1–3). Technology representation is further discussed in Methods.

**Table 1:** Scenario overview

Scenario	Description	Cost	Energy	Max cap	Growth rate
*Central case*					
NoNET	No BECCS and no DACCS, only afforestation allowed (+traditional CCS)				
NoDAC	No DACCS available, only BECCS and afforestation as NETs (+traditional CCS)				
DAC	Full NET portfolio: DACCS, BECCS and afforestation (+traditional CCS)	High	High	30 Gt/year	20%
*Sensitivity*					
LowCost	Lower end for cost estimates, both for DAC1 and DAC2	Low	High	30 Gt/year	20%
LowEnergy	Lower end for energy requirements, both for DAC1 and DAC2	High	Low	30 Gt/year	20%
gr15%	Lower annual growth rate for DACCS	High	High	30 Gt/year	15%
gr30%	Higher annual growth rate for DACCS	High	High	30 Gt/year	30%
Gt - gr20%	Maximum capacity for DACCS plants limited to 3 ${\mathrm{Gt}}_{{\mathrm{CO}}_2}$/year, 20% annual growth	High	High	3 Gt/year	20%
Gt - gr15%	Maximum capacity for DACCS plants limited to 3 ${\mathrm{Gt}}_{{\mathrm{CO}}_2}$/year, 15% annual growth	High	High	3 Gt/year	15%
Gt - gr30%	Maximum capacity for DACCS plants limited to 3 ${\mathrm{Gt}}_{{\mathrm{CO}}_2}$/year, 30% annual growth	High	High	3 Gt/year	30%
LowDisc	Low discount rate applied, close to 0% (see Supplementary Note 2)	High	High	30 Gt/year	20%
LowStorage	Limited storage availability, according to model characteristics (see Supplementary Note 3)	High	High	30 Gt/year	20%

This table shows the scenario names and key characteristics. Note that all sensitivities apply to the DAC scenario, i.e. the scenario where all three of afforestation, BECCS and DACCS are available

**Table 2:** Energy and cost assumptions, high and low scenario

Technology		Electricity [GJ/$${\mathrm{t}}_{{\mathrm{CO}}_2}$$]	Heat [GJ/$${\mathrm{t}}_{{\mathrm{CO}}_2}$$]	Cost [$/$${\mathrm{t}}_{{\mathrm{CO}}_2}$$]
DAC1	High	1.8^6^	8.1^6^	300^19^
	Low	1.3^17^	5.3^17^	180^17^
	Floor			100^17^
DAC2	High	1.1^22^	7.2^22^	350^6^
	Low	0.6^68^	4.4^68^	200^38^
	Floor			50^70^

This table shows the energy and cost assumptions for DACCS. Costs refer only to capital, labour and maintenance expenditure, while energy costs are determined endogenously by the models.

**Table 3:** Carbon budgets imposed, 66% probability

Climate target	Carbon budget 2016–2100^66^
2 °C	810 ${\mathrm{Gt}}_{{\mathrm{CO}}_2}$
0.5 °C	220 ${\mathrm{Gt}}_{{\mathrm{CO}}_2}$

This table shows the carbon budgets, defined as the cumulative CO_2_ emissions from 2016 to 2100, for the climate targets. These are associated with a 66% probability to keep the temperature below 2 and 1.5 °C, respectively.

We use two different IAMs, TIAM-Grantham, and WITCH. TIAM-Grantham is a bottom-up energy system model, with a detailed representation of technologies across sectors. WITCH combines a bottom-up energy sector description with a top-down macro-economic approach. Detailed information about both models can be found in Supplementary Notes 1–3.

According to the different model features, in TIAM-Grantham we have implemented both DACCS technologies, and explicit characterization of the recovery of waste heat, while in WITCH we have only included DAC1, fuelled by natural gas. We harmonize a range of input assumptions, which are later shown to be critical in determining model outcomes, regarding technology take-up levels and rates. In addition, DACCS costs, input and output flows and carbon budgets have been aligned between models. Cost reductions have been applied to model technical learning, with an exogenous learning rate in TIAM-Grantham and an endogenous one in WITCH. In both models, expansion constraints have been implemented as a 20% annual growth rate cap, according to historical benchmarks for technology development^28^). We impose the global maximum capacity at 30 GtCO_2_/year, in line with past NETs potential assessments^8–10,29,30^. BECCS and afforestation are included in both models (see Supplementary Note 4 and Supplementary Table 1).

We focus on scenarios consistent with 2 and 1.5 °C temperature increase limits. For each temperature scenario, we run three variant cases: one with only afforestation (NoNET scenario); one with afforestation and BECCS as per most published IAM-based studies (NoDAC); and a third one including DACCS in the mitigation portfolio (DAC). We perform sensitivity analysis on energy inputs, costs, growth constraints, discount rates and storage availability to test the robustness of model results. Table 1a summarizes all the scenarios.

### Central case scenarios

**Fig. 1:**
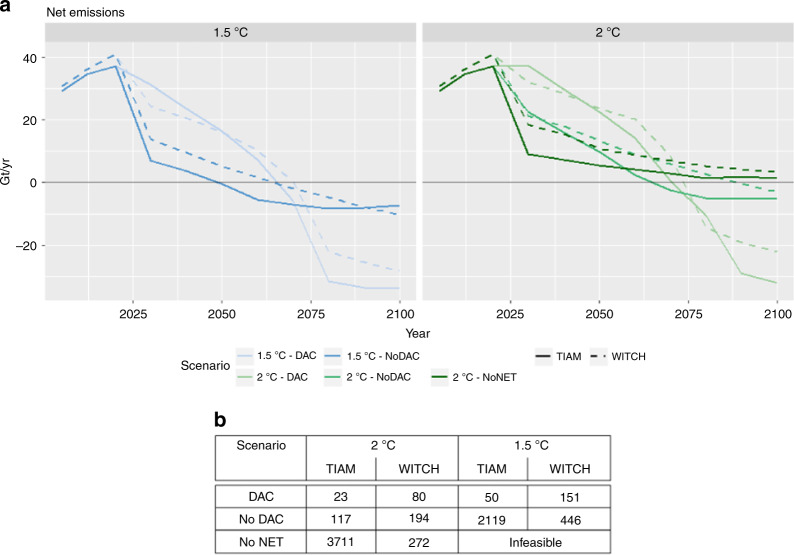
CO_2_ emission pathways and carbon price in 2030 in central case scenarios. CO_2_ emissions (**a**) are from fossil fuel burning only, while the carbon price (**b**) is expressed in USD per ton of CO_2_

DACCS has a significant influence on the mitigation pathway shape and the timing of peak emissions. Enabling DACCS as a technology option results in larger net emissions until around 2070, compensated by larger negative emissions thereafter in both models. In 2030 for a 1.5 °C scenario, DACCS allows emissions to increase by between 10 and 25–30 GtCO_2_. These emissions are similar to those of 2 °C scenarios without DACCS. Conversely, at the end of the century in 1.5 °C scenarios net negative emissions increase from 10 to 30 GtCO_2_/year. Although similar levels of CO_2_ removal are present in some existing IAM scenarios^3^, these are as large as current net emissions (Fig. 1).

In terms of policy cost, DACCS reduces the marginal abatement costs to achieve the climate target by between 60 to more than 90%. The 2030 global carbon prices with DACCS are below 100 and 200 $/tCO_2_ for 2 and 1.5 °C respectively (Fig. 1b). It should be noted that reaching a 1.5 °C target without relying on NETs is infeasible for both models, highlighting the key role NETs can play in increasing the feasibility of ambitious climate targets. While questions arise about the inter-generational equity of these pathways, as DACCS allows a reduction in near term mitigation effort in some energy-intensive sectors that are difficult to decarbonize, such as transport and industry. Higher emissions are allowed from these sectors when a full portfolio of NET options is available (see Supplementary Fig. 1), and oil still accounts for a large share of the primary energy Supplementary up to 2050 (see Supplementary Fig. 2 and Supplementary Note 5).

**Fig. 2:**
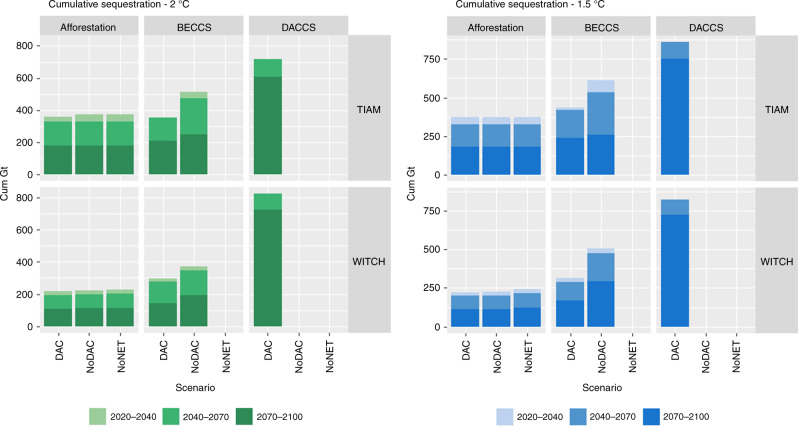
Cumulative sequestration of negative emissions technologies throughout the century in central case scenarios, with different temperature targets. The short (2020–2040), mid (2040–2070), and long-term role (2070–2100) of each strategy has been highlighted

DACCS appears to be a long-term mitigation measure, as it is largely deployed in the latter part of the century. Other NETs such as BECCS and afforestation are more evenly deployed over time, because they are more cost competitive (Fig. 2). Moreover, BECCS is able to produce carbon negative energy when it is deployed with new bioenergy capacity (as opposed to adding CCS to existing bioenergy capacity, which rather constitutes an energy penalty). By contrast, DACCS is treated by the models as a backstop to the exponential increase in the marginal abatement cost. It therefore substitutes for emission reductions more than other NET strategies, but does not provide a primary or secondary energy source for end-use services and requires additional primary energy supply.

In terms of the relation between different NETs, the total amount of CDR increases in line with the number of options that are available. There is some substitution: BECCS is reduced by 20–37% in DAC scenarios. Overall, though, both strategies are developed in tandem so as to avoid excessive specialization and consequently reduce risks and costs. Among the different DACCS options considered in TIAM-Grantham, the one based on solid amine sorbents (DAC2) appears the preferred one, especially with stringent mitigation. This can be related to residual emissions associated with burning natural gas for DAC1 heat provision (Supplementary Fig. 3a).

**Fig. 3:**
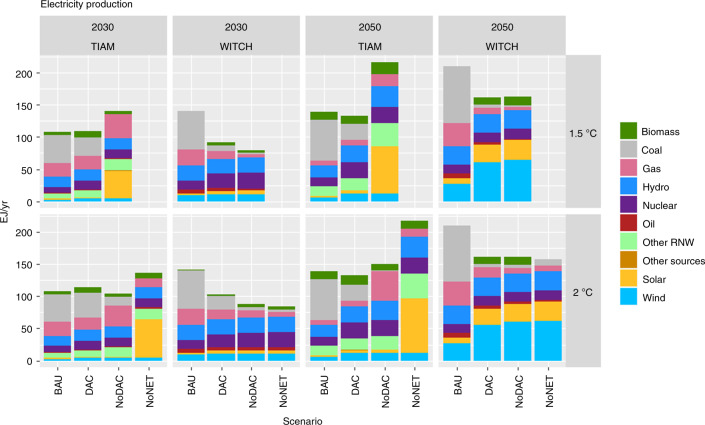
Electricity mix in 2030 and 2050 in central case scenarios, compared to the Business-As-Usual (BAU). BAU assumes no mitigation policy to be implemented from 2020 on: economic and population growth are calibrated according to Shared Socio-Economic Pathways 2 in both models

Figure 3 shows the implications for the electricity sector. DACCS long-term deployment reduces the need for a drastic decarbonization of the power sector. This is evident mainly from TIAM-Grantham results: in 2030 the electricity mix in DAC scenarios is not considerably different from Business-As-Usual, i.e. with no carbon constraint. Reaching a 1.5 °C target relying only on BECCS and afforestation (NoDAC) requires 50% of electricity generation to come from intermittent renewable sources by 2030, and a higher electrification across sectors, increasing the overall electricity demand (up to 70% more than in DAC scenarios). Moreover, DACCS enables delaying the phase-out of fossil-based electricity generation until after 2050.

### Sensitivity on techno–economic parameters

**Fig. 4:**
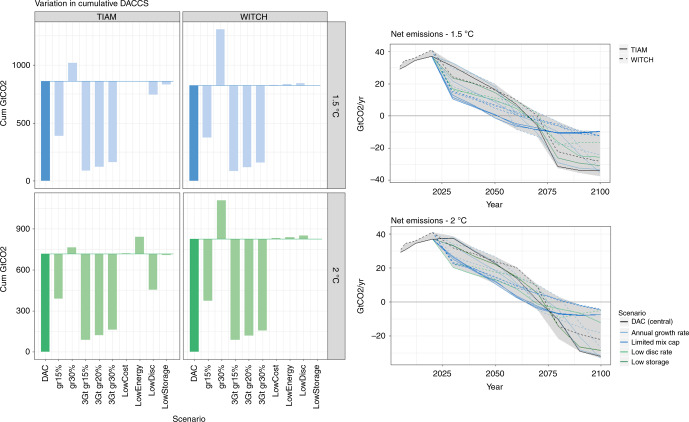
Sensitivity on key parameters: in **a** light green bars show the change in cumulative sequestration by DACCS with respect to the base case (i.e. *DAC* scenario, represented by the first bar) across all sensitivities; **b** shows the emission pathway. Sensitivities have been grouped into four categories to highlight the most influential factors: those related to annual growth rates, maximum capacity, discount rates and storage availability. Energy and cost sensitivities are not included due to the limited impact of these parameters

Energy requirements and investment costs for DACCS are currently uncertain. However, sensitivity analysis with the two IAMs shows that the influence of these parameters is limited in determining the overall DACCS deployment (Fig. 4a). Only in TIAM-Grantham, with a 2 °C warming, lower energy consumption leads to 125 GtCO_2_ more captured along the century. With a more stringent target (1.5 °C) this technology is deployed at the highest rate allowed, so that the only effect is on the share of the different DACCS technologies (see Supplementary Fig. 3b).

Conversely, expansion constraints are the key parameters determining DACCS deployment, especially for a 1.5 °C target (Fig. 4a). This is in line with the existing literature^8,9^ but creates a high degree of arbitrariness as there are no obvious land or natural resource constraints, though unclear whether the very rapid scale-up indicated here is feasible in reality (this aspect will be investigated in more in detail in the next section). Changing the annual growth rate limit to 15 and 30% results in ±450 GtCO_2_ cumulative capture across the century. The impact on net emissions is more marked when limiting DACCS capacity to 3 GtCO_2_/year. There is a strong reduction of residual emissions in the mid-term (Fig. 4b) and also repercussions on the energy sector (Supplementary Fig. 4a). DACCS cumulative sequestration is reduced by between 550 and 770 GtCO_2_, while BECCS captures ~20–50% more CO_2_ (Supplementary Fig. 4b).

Regarding inter-generational preferences, a lower time discount rate leads to earlier decarbonization in both models. This limits the need for large-scale carbon dioxide removal in later decades (Supplementary Fig. 5a). In TIAM-Grantham DACCS deployment is deeply affected, with cumulative sequestration decreasing by between 115 and 260 GtCO_2_, in the 1.5 and 2 °C targets respectively. However, in WITCH it remains almost unchanged, while BECCS capacity is reduced (see Supplementary Fig. 5b). In both models, the impact is less marked for a 1.5 °C target. In this case, all the options to remove CO_2_ are deployed at their maximum potential as there is less flexibility with regard to abatement timing.

With reduced CO_2_ storage availability, priority is given to DACCS with respect to other sequestration options, as its role cannot be substituted to tackle decentralized emissions. This results in less BECCS and CCS in both electricity and industrial sectors (Supplementary Fig. 6). As a consequence, electricity production relies more on renewables, and the resulting carbon price is approximately doubled (Supplementary Fig. 7).

Further information about sensitivity analysis can be found in Supplementary Note 6.

### Historical comparison for scale-up rates

Results discussed so far show that the main issue is not related to costs but to the rate at which DACCS can be ramped up. Indeed, model runs show DACCS maximum scale-up rate being an average 1.5 GtCO_2_/year, about twice the maximum rate of BECCS in similar mitigation scenarios^15^. Historical data can been used for benchmarking growth rates, so to help understand the feasibility of the foreseen diffusion pathways for DACCS. Typical historical rates are between 15 and 20% per year^28^, with the high end referring to modular technologies, rather than large scale complex facilities (see Supplementary Table 2). Indeed, DACCS technology allows smaller scale and relatively finer granularity compared to BECCS plants. The recent diffusion of solar PV at about 30%^31^ and forecasts for wind, expected to grow at 11% per year until 2050^32^ are aligned with the rapid scale-up of DACCS seen in our results.

**Fig. 5:**
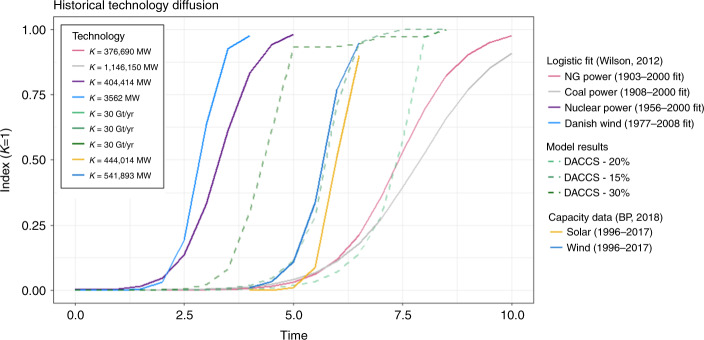
Comparison of DACCS up-scaling with historical technology diffusion. Past technology diffusion pathways are based on data from Wilson^33^ and recent statistics for solar PV and wind^35^. As these technologies have been diffusing in different years and with different scales (i.e. the extent *K* reached by the logistic profile), we have normalized the data indexing the capacity extent *K* to 1, and we have harmonized the starting year (*t* = 0), considering a unit time scale equal to one decade

Historical diffusion pathways have been modeled through logistic curves^33,34^. These allow the analysis of the entire life-cycle of technologies in terms of up-scaling, formative phase and saturation, thereby not focusing only on a limited time span of rapid growth. We compare DACCS growth profiles from our models with historical cases, including the logistic fit obtained by Wilson^33,34^ for different energy technologies and the recent diffusion of solar PV and wind capacity^35^ (Fig. 5). Even if DACCS deployment may appear incredibly rapid, from 1 to 30 GtCO_2_/year of removal in only 20 years, other technologies experienced similar patterns in the past.

This ambitious up-scaling pathway requires appropriate regulatory interventions and public acceptance: in practice, these could prove complex and challenging, in light of the experience of CCS has demonstrated limited societal support^36^. Currently, literature studies on NETs have focused extensively on the supply side of technology development, not addressing other key issues related to incentives for early deployment, niche markets and the regulatory framework^15^. Indeed, policy instruments and financial incentives supporting negative emission technologies are almost absent at a global scale, though essential to make NET deployment attractive, generating revenues linked to the carbon dioxide captured by these plants for instance^37^.

### Energy land water and material use

An other key parameter in our models is the maximum DACCS capacity in terms of GtCO_2_ captured. This is directly related to the number of plants that can be built, the energy input to operate them and the environmental impact in terms of land, water and material use. We investigate the implications of our scenarios on these aspects (results are shown in Supplementary Table 3).

If large-scale plants are going to be built^6^ (i.e. *DAC1* technology), capturing 30 GtCO_2_/year means installing 30,000 facilities. This is comparable with the cumulatively produced number of jet aircraft (21,000 in 1958–2007^33^) or natural gas plants (15,000 in 1903–2000^33^) built in the past. By contrast, considering Climeworks’s design^38^ (i.e. *DAC2*), around 30 million units could be required in operational stock by the end of the century. This is aligned to the world annual market for cars and commercial vehicles (73 million unit in 2017^39^).

**Fig. 6:**
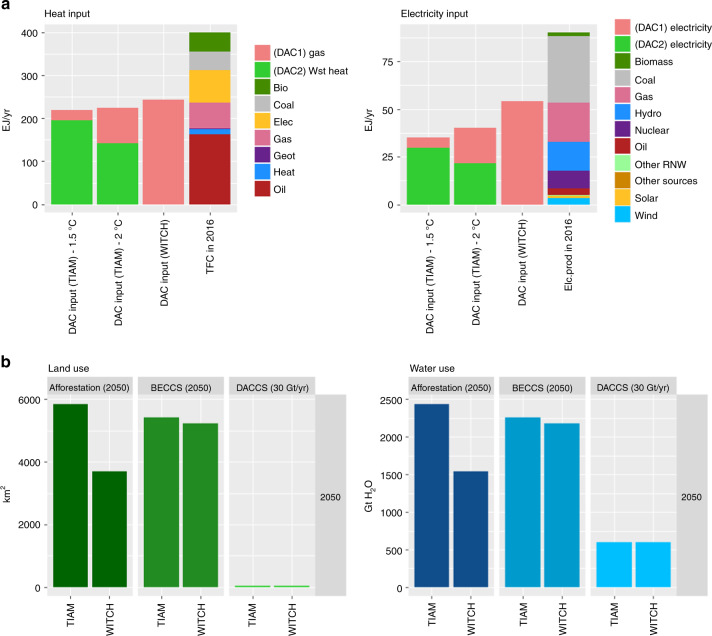
Impact of DACCS in terms of energy input, land and water use. **a** shows the energy input required to operate DACCS plants capturing about 30 GtCO_2_/year. Note that from TIAM-Grantham we have a differentiation among the two DACCS technologies, with different heat sources, while in WITCH we only have gas-fired DAC1 plants. Heat and electricity inputs are compared with the 2016 Total Final Consumption and electricity production respectively, as reported by the International Energy Agency^43^. **b** shows the amount of land and water used by DACCS plants to capture 30 GtCO_2_/year compared to BECCS and afforestation^5^, when these are deployed at the levels foreseen by the models in 2050

Past studies have investigated environmental implications of different CDR options^4,5,40–42^. Conversely to BECCS, there are fewer external constraints that may limit the deployment of DACCS a priori, as land and water use is significantly reduced compared to biological NETs (see Fig. 6b). Nevertheless, DACCS will have a significant impact on global energy provision.In 2100 it could require around 50 EJ/year of electricity, that is more than half of today’s total production^43^ (and about 10–15% of the global generation projected in 2100 by our models) and 250 EJ/year of heat, representing more than half of today’s final energy consumption globally^43^ (Fig. 6a). It should be noted that providing around 200 EJ/year of waste heat may have implications on the locations of DACCS plants: in order to avoid additional infrastructure and pipelines, these need to be co-located close to industrial facilities where waste heat is recovered, partially limiting the decentralization advantage of this technology. Nevertheless, this represents only one technological option to realize direct air capture: different sources of heat can be used according to site-specific availability, enabling a sufficient level of flexibility.

Moving to material use, we investigate the availability and the production process of the proposed chemical sorbents at large scale. Hydroxide solutions are currently obtained as a side-product of chlorine (Cl_2_) synthesis, which is the main market product. High DACCS deployment would reverse these roles, with sodium or potassium hydroxide becoming the most valuable output, thus disrupting the current market. Considering the sorbent replacement rate in DAC1 plants^18^, the current scale of the chlorine market of about 80 Mt/year^44^ will allow capture of 300–500 MtCO_2_/year. Moreover, sodium hydroxide is produced through an energy intensive chloralkali process, so that an additional 2.2–3.8 GJ of electricity is required per ton of CO_2_ captured. This is more than twice the electricity required to operate DACCS plants. For amine adsorbents, it is more difficult to investigate their impact as few technical details are available in the literature. As with amine sorbents employed for traditional CCS (MEA, monoethanolamine), synthesis is likely to start from ammonia and ethylene oxide. Their markets are about 177^45^ and 35^46^ Mt/year, respectively, and they both come from fossil fuel feedstock. Based on MEA production process^47^ with a similar replacement rate to DAC1 plants, 15–26 Gt/year of ammonia and 3–5 Gt/year of ethylene oxide will be required. These are approximate calculations, as the long-term sorbent stability is still unknown^48^, but they provide a starting point to understand the challenges behind such a scale-up of DACCS. These comparisons highlight the requirement for more detailed process-scale operational data and techno–economic assessments for policy makers to understand the full costs and potential of DACCS.

### Carbon cycle feedback

An important issue to be considered is the behaviour of natural carbon sinks to negative emissions, that may hinder the effectiveness of NETs^49–52^. Past studies using Earth System Models^51^ estimate that removing 491 GtCO_2_ from the atmosphere over a period of 30 years (16 GtCO_2_/year GtCO_2_/year) or 10 years (49 GtCO_2_/year) would result in 51 or 95 GtCO_2_ outgas emissions from the oceans respectively. From our scenarios, DACCS is foreseen to remove between 16 and 30 GtCO_2_/year over the period 2070–2100. This implies that a significant fraction (from 10 to 19%) of the carbon removed would be released back to the atmosphere from the oceans, requiring an additional removal of 1.7 to 9.5 GtCO_2_/year to meet the same carbon budget. Indeed, accounting for carbon cycle feedbacks within IAMs has been shown to decrease the attractiveness of CDR^8^.

### What if DACCS fails to deploy at scale?

**Fig. 7:**
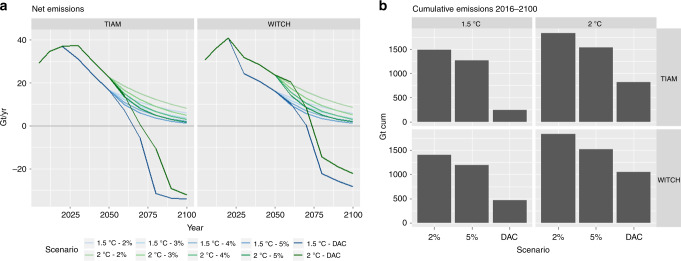
Emission pathway and cumulative emissions in DACCS failure scenarios. The left panels **a** shows the emission pathways of the original DACCS scenarios and those with no DACCS and exogenous emission reductions between 2 and 5%. The right panels **b** show the 2016–2100 cumulative emissions of CO_2_. In this case, carbon emissions for WITCH include both fossil burning, industry and land use

Given the still-significant uncertainties about NETs feasibility, we seek to understand the impact of relying on NETs to reach mitigation targets, only to find out later that they do not perform as anticipated. The emission pathways shown in Fig. 7a follow DAC scenarios up to 2050. After that date we assume that no DACCS (and no BECCS/afforestation) is going to be installed, and we simply apply a set of exogenous emission reduction rates varying between 2 and 5% per year. These are aligned with historic emission reduction rates achieved in some countries for consecutive years^53^, and are meant to simulate the ambitious efforts in the absence of carbon removal strategies. Nevertheless, these decarbonization rates could conceivably be exceeded, so they are used purely as an illustrative range, subject to high uncertainty. Not having NETs after mid century increases cumulative emissions between 600 and 1200 GtCO_2_. Considering a transient climate response to cumulative emissions (TCRE) to be in the range of 0.8–2.4 °C/TtC^54^, not having DACCS leads to a temperature overshoot by the end of the century of 0.15–0.8 °C, according to the range of emission reduction rates investigated. That is, targeting 1.5 °C with DACCS and then not having it would likely lead to a warming of 2 °C and more.

It should be noted that this temperature change estimate does not account for any additional reduction in mitigation effort that the assumption of future emissions removals could have, based on moral hazard considerations. These deterrence effects, that result in NETs substituting for emission reductions rather than supplementing them, include also rebounds and side-effects (e.g. land-use change, use of captured CO_2_ in enhanced oil recovery) and may result in a larger temperature increase by the end of the century^55^. It is therefore essential that policy makers make informed decisions so as to undertake early mitigation with a full consideration of these risks^56^, as well as an appropriate reflection of the economic costs, which future generations can reasonably bear.

## Discussion

Our analysis shows that in theory DACCS can be an enabling factor for the Paris Agreement objectives: it allows their achievement at lower costs, by more-than-halving carbon prices in 2030. This follows from the reduction of near-term mitigation in exchange for higher long-term atmospheric CO_2_ removal by DACCS technologies, which includes a delayed phase out of fossil fuels from the power sector until after 2050.

The analysis also highlights the clear risks of planning a long-term mitigation strategy on the assumption that DACCS will be available and can scale up at speed. First, we find that the speed of DACCS scale-up is the greatest sensitivity in its ability to remove CO_2_ and ease the mitigation burden on the energy system. A failure to achieve this scale-up risks locking the energy system into fossil fuels and making the long-term temperature goal much more costly and less feasible. Second, whilst the analysis posits that the indicated DACCS scale-up may not be unprecedented when compared to some other technologies (notably jet aircraft), it highlights several potential barriers to deployment. These include the chemical pollutant implications of sorbent manufacture at vast scales, as well as a requirement to use around a quarter of global energy demand to provide power and heat for DACCS technologies by the end of the century. Therefore, it is important to include DACCS within a diversified mitigation portfolio in low carbon scenarios, together with other CDR strategies, demand-side and lifestyle measures^36,57^. We recommend that further analysis undertake a full life-cycle assessment of DACCS, in order to understand how its deployment drives energy demand for sorbent manufacture, as well as energy and material demand such as cement and steel for DACCS equipment. Finally, the scale-up of DACCS indicated would only be possible with an appropriately comprehensive CO_2_ transport and sequestration infrastructure, as well as a strong regulatory and planning framework and public acceptability. Given the challenge of addressing these barriers, it is instructive to highlight that failure for DACCS to materialise, when previously planned for, could lead to up to 0.8 °C of warming overshoot.

Given the limits of IAMs in accounting for such risks, our preliminary considerations should be supplemented with further analysis and an appropriate theoretical framework^58–60^ accounting for interactions between technologies, society and political power, so to highlight optimal mitigation strategies in light of the uncertainty of future DACCS deployment and governance.

The potential economic benefit from DACCS, whilst considerable, must be weighed up against a fuller analysis of its technical viability and scale-up potential, in light of all of the challenges highlighted above. Given the urgency and importance of achieving the Paris Agreement goals, we recommend to policy makers that they support an acceleration in development and deployment of DACCS, but without easing near-term mitigation efforts^60^, so as to manage the risk of DACCS underperformance or failure.

## Methods

### Study design

This study is based on modeling long-term mitigation scenarios using two IAMs: TIAM-Grantham^61,62^ and WITCH^63,64^. We model climate targets by imposing a carbon budget over the period 2016–2100 equal to 810 and 220 GtCO_2_, consistent with 2 °C and 1.5° warming respectively^65,66^. In addition to the sequestration options already implemented in the models (CCS in electricity and industrial sectors, afforestation and BECCS), we include also a technology to capture CO_2_ directly from the air and store it under the ground, namely DACCS. We differentiate costs and energy inputs according to the literature, including cost reduction over time due to technical learning, and we implement growth constraints to model a feasible deployment rate within the models.

In addition, an expert elicitation was conducted to better understand the potential of this new technology from the perspective of specialists currently researching in this field, given the fragmentary and limited literature available on this topic.

The inter-model study design ensures that our results are robust across model uncertainties, as the IAMs adopted have complementary characteristics, combining a detailed bottom-up energy system model (TIAM-Grantham) and a hybrid model (WITCH) with a top-down representation of macro-economic variables. To test robustness against parametric uncertainty, we perform a sensitivity analysis on cost and energy parameters, annual growth rates and maximum capacity levels, as well as investigating the impacts of limited CO_2_ storage capacity and a lower time discount rate. A detailed description of the IAMs adopted can be found in Supplementary Note 1, together with further information about time discount rates (Supplementary Note 2), storage availability (Supplementary Note 3) and CCS/BECCS cost assumptions (Supplementary Note 4).

In addition, it should be noted that WITCH includes carbon emissions from fossil fuel and industry (FFI) and from the land use sector, while TIAM-Grantham only considers FFI emissions. In both 1.5 and 2 °C scenarios, net cumulative land-use emissions over the century in WITCH are close to zero.

### Modeling DACCS technologies

Looking at the state of the art for capturing CO_2_ directly from the air, research has focused on the application of aqueous hydroxide solutions (DAC1) and amine-modified solid sorbents (DAC2). These two technologies are employed in the first DACCS plants built around the world, and therefore closer to being commercially available; other innovative solid sorbents have not been included as they are still at an earlier research stage. We characterize these two processes in terms of cost and energy inputs: while in TIAM-Grantham we implement both DAC1 and DAC2, given the technological details allowed by this model, in WITCH we only include the former.

DAC1 technology refers to the first plant design proposed by the American Physical Society (APS) in 2011^6^ and further developed through the work of Keith and Holmes^17,20^, together with the company Climate Engineering. It is based on a two-loop hydroxide-carbonate system (NaOH-CaOH or KOH-CaOH), with a plant reference size of 1 Mt/year of captured CO_2_. This design involves large energy needs, mainly high-temperature heat for sorbent regeneration that can be provided by burning natural gas: electricity requirements range between 1.3^17^ and 1.8^6^ GJ/tCO_2_, and heat input between 5.3^17^ and 8.1^6^ GJ/tCO_2_. An additional CCS unit is needed to capture the CO_2_ emitted when burning natural gas, with a capture efficiency equal to 95%, similar to natural gas combined cycle (NGCC) plants with oxyfuel CCS already implemented in both models.

The second option, DAC2, is based on amine-functionalized adsorbents^67^, currently under development by Climeworks in Switzerland and Global Thermostat in the US. Few technical details are available, due to commercial confidentiality. Solid sorbents have lower energy consumption, given the lower regeneration temperature (electricity: 0.6^68^–1.1^22^ GJ/tCO_2_, heat: 4.4^68^–7.2^22^ GJ/tCO_2_). Significant cost reductions are expected in the future, both because of its modular design and the low level of technological maturity. As the low-temperature heat may be provided through heat recovery, the energy input has been modeled as a waste heat stream coming from industrial processes and low-carbon power plants. This allows a reduction of the overall cost of capture, as energy needs appear to be one of the main cost components for DACCS plants. The main drawbacks for DAC2 are degradation and stability issues related to the amine materials, leading to higher operational expenditure due to sorbent replacement.

We use scientific papers to determine the higher cost bounds, and estimates from private companies running the first demonstration plants to define the low cost scenario and the floor value, as summarised in Supplementary Table 4. The operating expenditure reported does not include energy costs, as these are determined endogenously by the models.

For DAC1, Mazzotti’s optimization on the APS design^19^ provides the high cost scenario, with an overall cost around 300 $/tCO_2_ (excluding energy), while the latest paper by Keith and Holmes issued in June 2018^17^ gives both the low cost estimates (180 $/tCO_2_) and the target for future cost reduction, around 105 $/tCO_2_. Both these references provide a detailed cost breakdown, enabling specification of CAPEX and OPEX, as well as individual cost components. Combined with low-cost renewable energy, for example in North Africa, this cost can be even lower^69^.The high floor cost reflects DAC1 limited potential for future cost reductions, as this technology is based on processes and equipment already well-known and developed in other sectors.

Capture plants based on amine-modified adsorbents (DAC2) are characterized by higher operational costs due to frequent sorbent replacement because of degradation. As no exhaustive cost assessments for this technology are publicly available, we start from the APS^6^ estimate for the overall capture cost (350 $/tCO_2_ without energy), but switch the fraction allocated to OPEX and CAPEX (74 and 26%, respectively), to reflect the specifics of this technology option. We use the same percentage allocation of overall costs between CAPEX and OPEX for the low and the floor cost estimates. The low cost scenario is defined according to Climeworks’ estimate^38^ (200 $/tCO_2_), while the floor cost of 50 $/tCO_2_ is that claimed by Global Thermostat^70^.

Floor cost identifies the long-term target that could be achieved in the future, and is used to set a lower bound for cost reduction. In both IAMs cost reduction is implemented to model technical learning over time, with an exogenous rate in TIAM-Grantham (6% per year, aligned with historical cases in energy and chemical sectors^71^) and an endogenous learning curve in WITCH (learning rate equal to 0.06, the same used for natural gas plants with CCS).

Information on techno–economic parameters are summarized in Supplementary Table 4 and Supplementary Fig. 8.

### Waste heat recovery in TIAM-Grantham

As explained earlier, waste heat can be employed to operate amine-based plants and it is already implemented in Climeworks’ and Global Thermostat’s pilots. We include this aspect in TIAM-Grantham, given its detailed technological representation of the energy system. We define a new commodity to model the waste heat recovered both from energy-intensive industrial processes (e.g. pulp and paper, iron and steel production) and from the power sector. As the waste heat potential will be limited, two distinct processes to represent amine-based DACCS have been defined, namely DAC2 and DAC21 (see Supplementary Fig. 8): heat is supplied through waste heat to the former, and through heat made on purpose to the latter. They are characterized by same economic and technical parameters, so that the model is free to install as much capacity of amine-based plants deploying DAC21, with no constraints deriving from the availability of the waste heat commodity. From our results DAC21 is almost never installed, as it is not economic to produce heat solely to fuel DACCS plants

To determine the recovery potential of industrial waste heat, we use analysis by Ecofys^72^, which examines a number of heat-intensive industrial sectors, such as refineries, iron and steel, ceramics, glass, chemicals, food, drink and pulp and paper industry. Referring to this data, we identify the sectors where the new commodity may be applicable and we define the production of waste heat as a fraction of the energy input, with this recovery factor *RF* changing according to the industry, as shown in Supplementary Table 5. As in our recovery process the sink is represented by the amine regeneration step, taking place at temperatures below 120 °C. Only industrial heat flows with temperature equal to or higher than 140 °C examined in the report have been taken into account. We put a cap on the capacity of industrial processes connected with waste heat, to avoid the model over-installing them only to provide the heat needed by DACCS plants. The constraint is based on the capacity installed in a baseline scenario without DACCS, with a 2 °C mitigation target.

Waste heat was also allowed to be sourced from some low-carbon power plants, namely nuclear and concentrated solar power. In the first case, waste heat can be recovered from the steam cycle in addition to the primary electricity output, while in the latter case, heat is produced as in Combined Heat and Power (CHP) units, slightly reducing the primary electricity outflow.

For nuclear plants, electricity generation efficiency is about 33%, meaning that about two-thirds of the energy in the fuel is lost and dissipated to the environment as heat. The temperature of the dissipated heat is sufficiently high to be supplied to amine-based DACCS plants, therefore in TIAM-Grantham all existing nuclear processes have been specified to include waste heat as an auxiliary output commodity (in addition to electricity). The overall efficiency of cogenerative plants is usually about 80%, including an electrical efficiency *η*_*el*_ = 35% and a thermal efficiency around 45%^73^. Therefore, for each PJ unit of electricity in output, 1.3 PJ of waste heat may be obtained (*RF*_*nucl*_).

1 $$RF_{nucl} = \frac{{Q_{wst}}}{{W_{el}}} = \frac{{\eta _{th}}}{{\eta _{el}}} = \frac{{0.45}}{{0.35}} = 1.3$$

Conversely, a new process has been defined to differentiate Concentrated Solar Power (CSP) plants where heat can be recovered (CHP mode) from those only producing electricity. This technology will use a steam power plant with back-pressure configuration, that means the electricity output is reduced, increasing the temperature at the steam turbine outlet to be sufficiently high to allow heat recovery at the condenser. Therefore, CHP solar plants (CSP–CHP) will have lower electrical efficiencies, going from *η*_*el*,*CSP*_ = 20% to *η*_*el*,*CSP*–*CHP*_ = 15%^74^. As costs are allocated to a reduced electricity output, this technology will be treated as more costly in the model with respect to traditional CSP plants. The recovery factor *RF*_*CSP*_ is computed considering a recovery efficiency for the heat output at the condenser *η*_*t*_ equal to 80%^74^.

2 $$RF_{CSP} = \frac{{Q_{wst}}}{{W_{el}}} = (\frac{1}{{\eta _{el,CSP - CHP}}} - 1)\cdot \eta _t = 4.53$$

### Comparison with historical technology diffusion

We compare DACCS deployment pathways from our results with other historical technology diffusion in order to understand its feasibility. We start from the work done by Wilson^33,34^ to model scaling dynamics across a range of technologies. This describes the common s-shaped growth profile with logistic function parameters, starting from historical time series data on refineries, power plants (nuclear, coal, gas, wind), jet aircraft, cars and lightbulbs. As these studies demonstrate a consistent relationship between the extent and the rate of scaling, measured in terms of cumulative total capacity, we use the same dataset as a benchmark to validate our projections for DACCS uptake up to 2100. Given that each technology is characterized by a different extent (i.e. maximum level of capacity reached during saturation phase) and their up-scaling takes place during different time periods, we normalize the data, indexing the capacity extent *K* to a value of 1, and consider a general timescale, with *t* = 0 representing the starting year of diffusion (first unit installed), and each unit of time corresponding to one decade (*t* = 1 coincides with 10 years later than the starting one).

In addition to this historical data, mainly referring to the 20th century, we have included also time series data about the recent rapid growth of low-carbon technologies, such as solar PV and wind, using British Petroleum statistics up to 2018^35^.

### Impact assessment for DACCS technologies

According to the assessment made by Smith^5^ on physical and economic impacts of large scale NETs deployment, one of the advantages of DACCS is the reduced footprint with respect to BECCS or afforestation, in terms of water and land use and sustainability implications (values adopted are summarized in Supplementary Table 6). Considering a sustainable bioenergy supply for BECCS plants, we have explicitly imposed a limit of 200 EJ/year in TIAM-Grantham, according to a past assessment^75^, that results within the range of models used in RF1.9 scenarios^66^. In WITCH this limit is not explicit, but it is never hit. Differently, there are no external constraints that may limit the deployment of DACCS a priori, such as material scarcity or environmental footprint, while a major issue is likely to come from the provision of chemical sorbents. Moreover, DACCS offers an additional location flexibility so that the capture facility can be placed closer to geological sequestration sites, avoiding long pipelines.

According to Smith et al.^5^, the land footprint related to BECCS power plants is between 270 and 1636 m^2^ to capture 1 tonne of CO_2_ per year, depending on the type of feedstock used as input fuel: the lower end corresponds to purpose-grown crops, while more land is required for agricultural residues due to their lower energy yield. Similar values can be applied also to afforestation, as they are both biological sequestration strategies. For DACCS, it is difficult to quantify the amount of land needed, given that there are currently so few pilot plants and each of the proposed designs require different plant components. Generally, the amount of land needed is limited to the one for building plant facilities, due to the low risk of build-up of CO_2_ deficient air around the capture plant^21^. Amine-based plants would require around 0.05–0.1 m^2^/tCO_2_/year^12,13^, while those using hydroxide solutions around 1.5 m^2^/tCO_2_/year^6^. The land footprint could increase considerably if solar PV panels or wind turbines were used to provide energy required, though unproductive land supplying minimal ecosystem services could in principle be allocated as sites.

Estimates for water requirements to remove one tonne of carbon by DACCS are about one order of magnitude or more lower than for BECCS plants, as BECCS demands water both for growing crops and feedstock and for operating the CCS module. Again, similar values can be applied to afforestation^5^. Water loss may represent a concern for some DACCS systems: aqueous systems are prone to evaporation, leading to a consumption of about 5 to 13 tonne of water per each tonne of carbon dioxide captured during normal operation, depending on humidity and air temperature^14,76^. Developers of amine-based plants do not mention water use as a source of concern, but other solid sorbents tested for DACCS application^24^ may require up to 20 tonnes of H_2_O, so that this has been taken as the high end for our assessment.

Considering the material use, the production of hydroxide sorbents is not straightforward, as a large amount of energy is required to synthesize sodium hydroxide (NaOH) and potassium hydroxide (KOH) (13.3 GJ/t_*NaOH*_ and 7 GJ/t_*KOH*_^77^ respectively). Moreover, currently these chemicals are obtained as a side product from chlorine (Cl_2_) production process, through electrolysis of sodium chloride and potassium chloride solutions respectively. In the future their respective roles may be reversed, with hydroxides being the most valuable outputs should DACCS reach such significant deployment.

According to the APS report^6^, there is a loss of NaOH solution during each capture cycle, as it remains partly entrained in the CO_2_-depleted air leaving the absorber. Considering the detailed mass balance provided by Baciocchi^18^, the make-up rate of sorbents is between 0.17 and 0.29 tonne per each tonne of CO_2_ captured, according to the different plant designs proposed. Note that this reference has been taken as a worst-case scenario for such technology. Currently the market for Cl_2_ is about 76.8 Mt/year^44^, which would allow about 300–500 MtCO_2_ being captured using *DAC1* plants. Therefore, the decision to limit DACCS maximum capacity to 3 and 30 GtCO_2_/year means scaling the current hydroxide market by a factor of 10 and 100 respectively. As a comparison, the current market for KOH and NaOH are about 0.8 and 80 Mt/year, respectively. Finally, it must be noted that chlorine gas would be the main byproduct of hydroxide production raising concerns about its handling, since it is a highly poisonous gas and a potential ingredient in chemical warfare.

When moving to amine-based adsorbents, it becomes much more difficult to investigate their production and the need for sorbent replacement during the capture process as very few technical details are available in the literature. Therefore, we analyze the production process of polyethanolamines (mainly MEA, monoethanolamine) which are generally employed to capture CO_2_ from power plants, through post-combustion CCS processes. In this case, the synthesis starts from ammonia and ethylene oxide, requiring about 3.2 and 0.64 tonnes respectively per each tonne of MEA produced^47^. Both of them come from fossil fuel precursors, mainly oil and natural gas, although ethylene can also be made from biomass. It is an endothermic process, so no heat input is required to sustain the reaction, beside the energy costs for running the production plant itself, which can be neglected. There is no robust information about the replacement rate for amine-based adsorbents in *DAC2* plants, but it is likely to be considerable given the degradation of amine materials due to oxidation and steam exposure^48^. As a conservative approach, we consider a similar make-up rate to hydroxide solutions, resulting in 15 to 26 Gt/year of ammonia (current market: 177 Mt/year^45^) and 3 to 5 Gt/year of ethylene oxide (current market: 35 Mt/year^46^).

All these values are summarized in Supplementary Table 3.

## Supplementary information

Supplementary Information

## Data Availability

The data that support the findings of this study are available from the authors on request.
