# Supplementary material for: An inter-model assessment of the role of direct air capture in deep mitigation pathways
Source: Nat Commun. 2019 Jul 22;10:3277. doi: 10.1038/s41467-019-10842-5 (PMC6646360; doi:10.1038/s41467-019-10842-5)
Supplement: Supplementary file 1 — Supplementary Information [file 41467_2019_10842_MOESM1_ESM.pdf]

# An Inter-Model Assessment of the Role of Direct Air Capture in Deep Mitigation Pathways

**Giulia Realmonte, *et al.***

## ABSTRACT

## Supplementary Information

**Supplementary Figure 1. Carbon Emissions across various sectors in TIAM-Grantham, with different NET strategies implemented.**

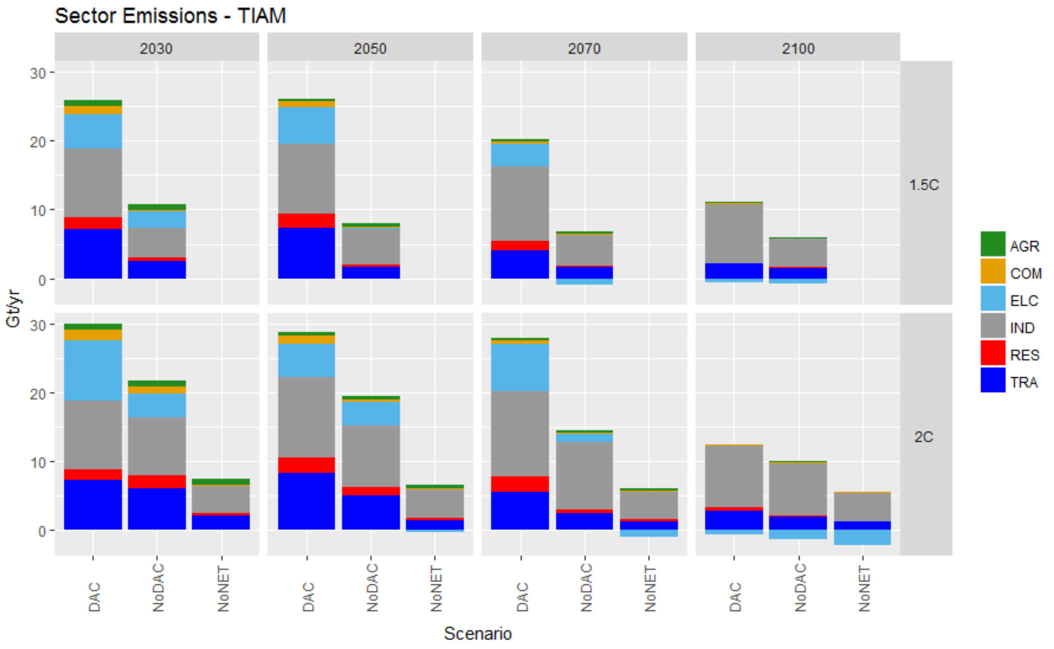

**Supplementary Figure 2. Total Primary Energy Supply (TPES), with different NET strategies implemented** compared to the Business-As-Usual (BAU) scenario, that assumes no policy to be implemented from 2020 on.

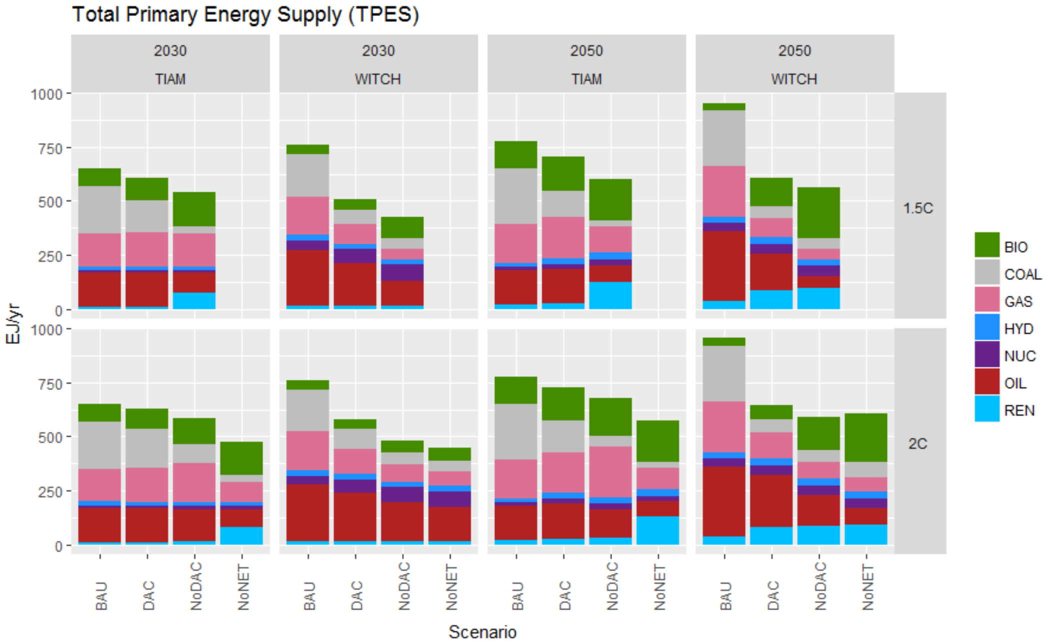


**Supplementary Figure 3. DACCS technology differentiation in TIAM-Grantham across base case scenarios (a) and sensitivities (b).**

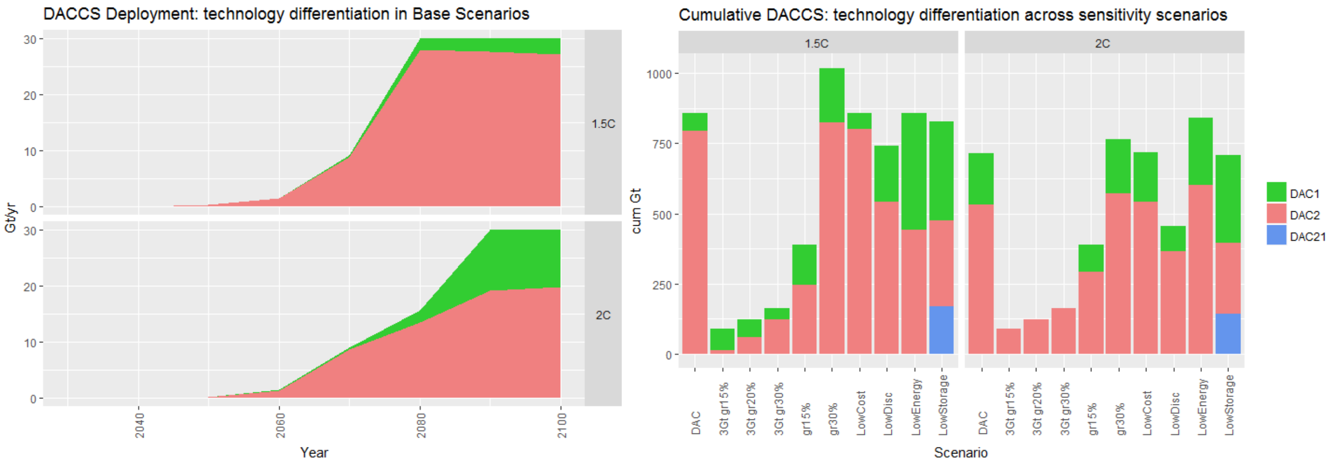


**(a)** DACCS Deployment in base scenarios

**(b)** Cumulative DACCS across sensitivity scenarios

**Supplementary Figure 4. Impact of a reduced DACCS capacity limit on other NETs deployment (a) and on the energy sector (b).**

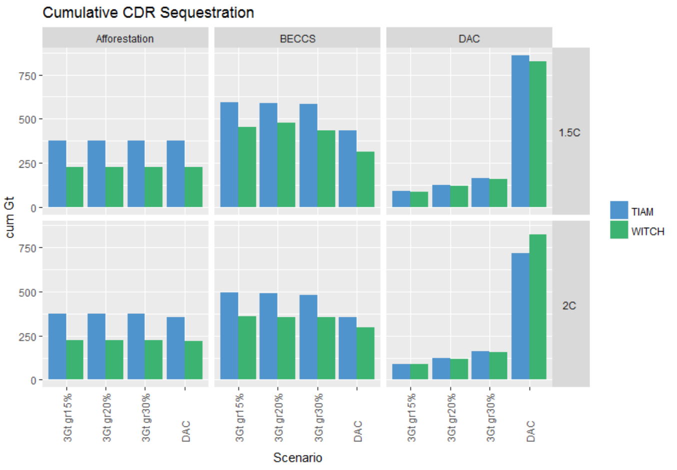


**(a)** Cumulative CDR sequestration along the century

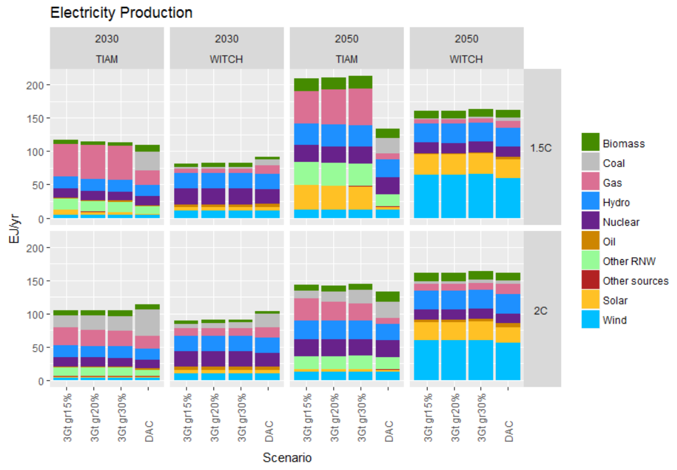


**(b)** Electricity Mix in 2030 and 2050

**Supplementary Figure 5. Impact of a low time discount rate on net emission (a) and CDR deployment (b).**

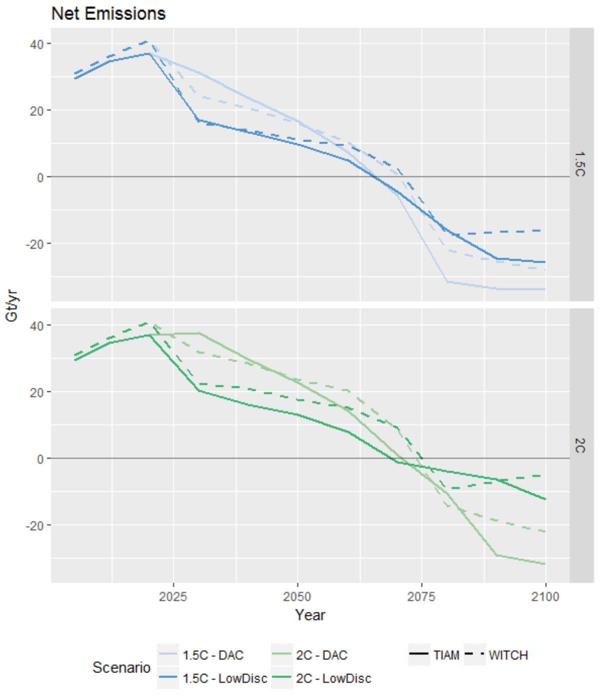


**(a)** Net Emission Pathways

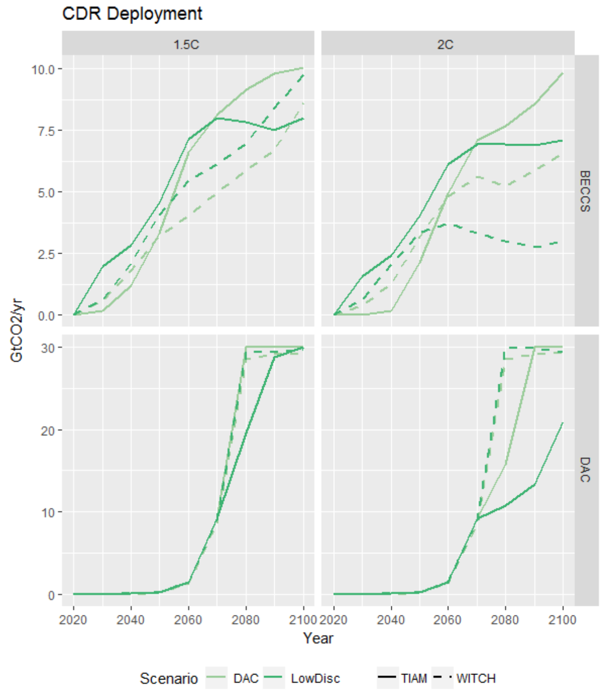


**(b)** BECCS and DACCS deployment

**Supplementary Figure 6. Impact of a reduced storage availability on net emission (a) and CDR deployment (b).**

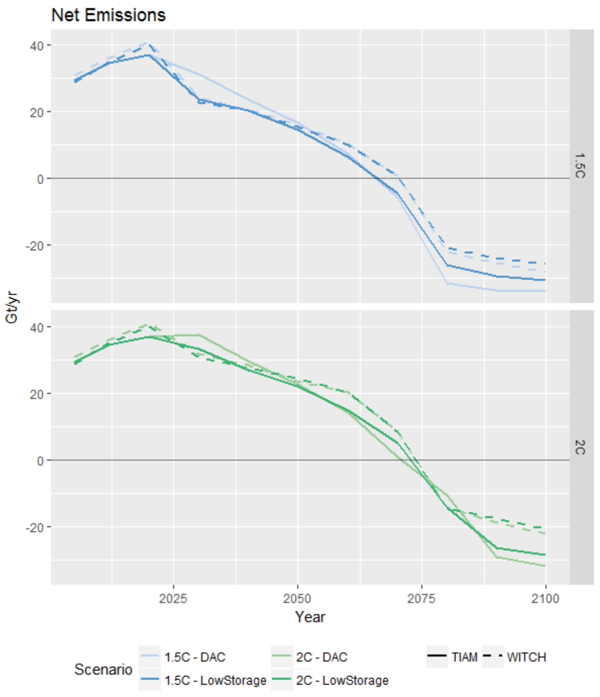


**(a)** Net Emission Pathways

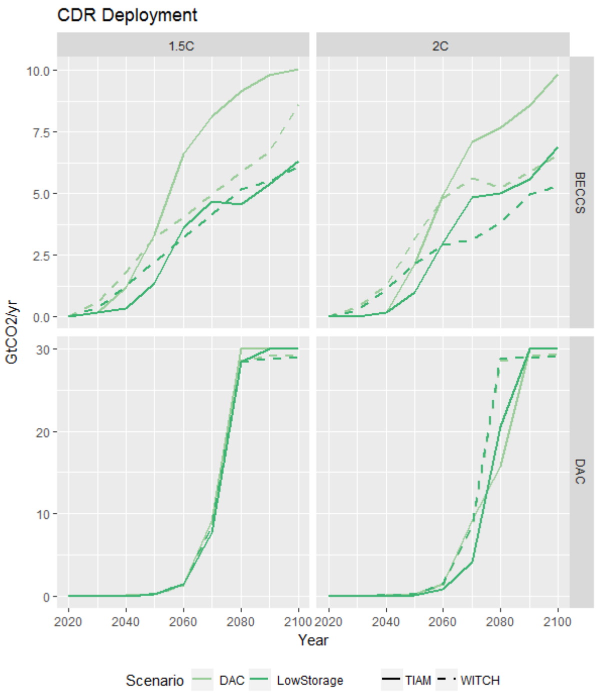


**(b)** BECCS and DACCS deployment

**Supplementary Figure 7. Electricity mix in 2030 and 2050 with a lower discount rate (0%) and a limited storage availability.**

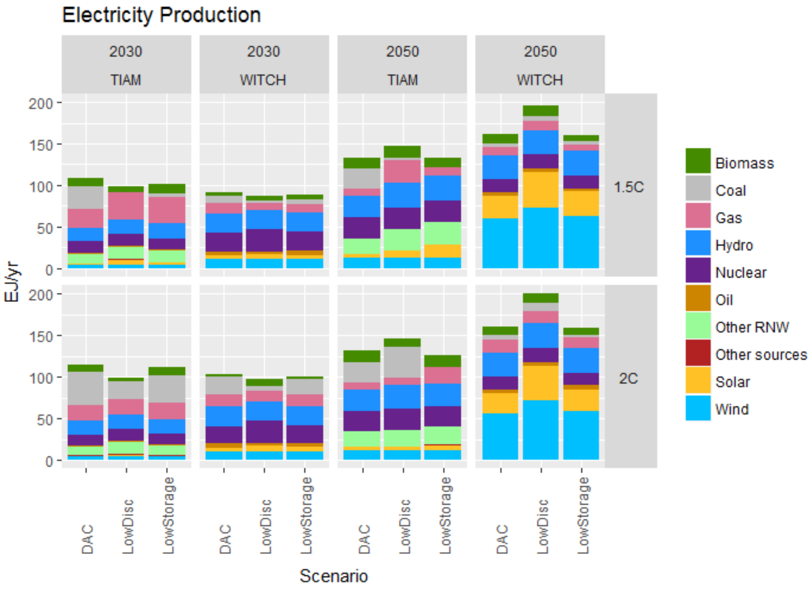

**Supplementary Figure 8. Modeling DACCS Technologies: input and output flows and energy requirements**

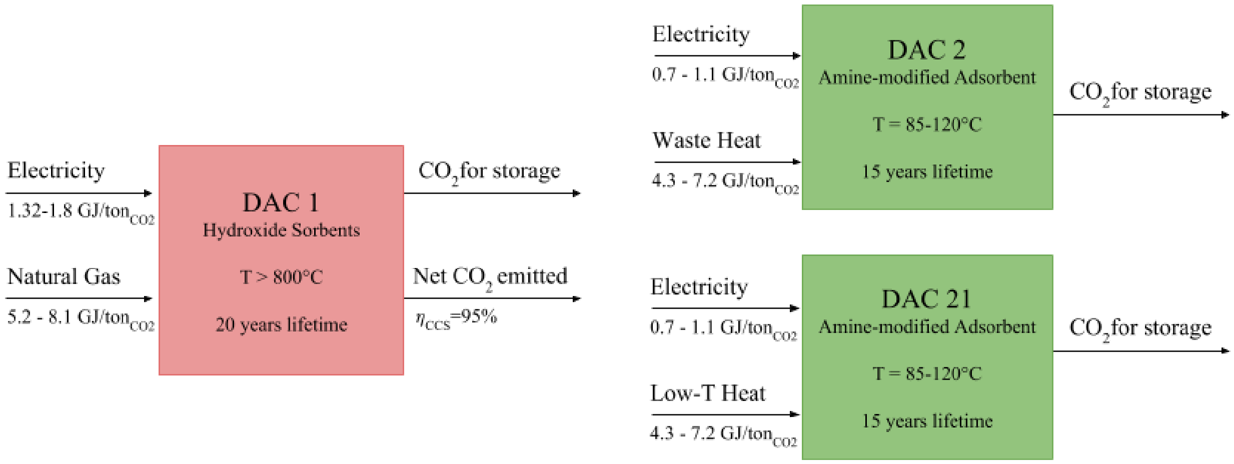

**Supplementary Table 1. CCS and BECCS Cost Assumptions in TIAM-Grantham and WITCH.**
*Gas-based plants with oxy-fuel CCS are implemented only in TIAM-Grantham, therefore their cost is estimated considering the same percentage difference between price of post-combustion and oxyfuel capture processes for coal plants.
** Differentiation between bioenergy power plants based on biomass gasification or direct combustion is implemented only in TIAM, while WITCH consider one single technology.

| **Technology** | | | **CAPEX** start | $[\$/kW]$ floor | **OPEX** start | $[\$/kW]$ floor | **Efficiency** $[\%]$ | |
|---|---|---|---|---|---|---|---|---|
| CCS | Coal | pre-combustion | 2740 | 1310 | 79 | 69 | 34 | TIAM/WITCH |
| | | post-combustion | 2727 | 1310 | 104 | 69 | 34 | TIAM/WITCH |
| | | oxy-fuel | 2896 | 1310 | 74 | 69 | 33 | TIAM/WITCH |
| | Gas | post-combustion | 1342 | 668 | 50 | 44 | 48 | TIAM/WITCH |
| | | oxy-fuel* | 1426 | 668 | 50 | 44 | 47 | TIAM |
| BECCS ** | | gasification | 2458 | 1826 | 77 | 56 | 28 | TIAM |
| | | direct combustion | 3281 | 2566 | 93 | 62 | 28 | TIAM |
| | | general techn. | 3288 | | 80 | | 28 | WITCH |

**Supplementary Table 2. Annual growth rate of multiple technologies in the past and recent deployment of low-carbon energy sources (solar PV and wind).**

| Source | Technology | Growth Rate | | |
|---|---|---|---|---|
| Iyer, 2015 [28] | Washing Detergent (as a substitute for soap) | 24% | USA | 1945-1960 |
| | Wind energy | 20% | Denmark | 1977-2008 |
| | Nuclear | 19% | France | 1977-1997 |
| | Cars (as a substitute for horses) | 18% | USA | 1900-1980s |
| | Nuclear energy | 11% | global | 1956-2000 |
| IEA, 2015[78] | Solar PV | 40% | global | 1999-2017 |
| SolarPower Europe, 2018[31] | Solar PV | 33% | global | 2012-2017 |
| IRENA, 2018[32] | Offshore wind | 11% | global | 2018-2050 |

**Supplementary Table 3. Implication of DACCS deployment at 30 $GtCO_2$/yr: number of plants, energy input and material use.**

**Number of Plants:**

| Plant Design | | Plant Unit Size | N°Units at 30$GtCO_2$/yr | Term of Comparison |
|---|---|---|---|---|
| large-scale plant | DAC1 | 1 $MtCO_2$/yr[11] | 30,000 | 21,000 jet aircrafts (1958-2007)[33] |
| | | | | 15,000 natural gas plants (1903-2000)[33] |
| modular design | DAC2 | 900 $tonCO_2$/yr[38] | 33 millions | 73 millions commercial cars (2017)[39] |
| | | | | 97 millions commercial vehicles (2017)[39] |

**Energy Input:**

| | Scenario | DAC Input [EJ/yr] | Term of Comparison | | [EJ/yr] | |
|---|---|---|---|---|---|---|
| **Heat** | TIAM - 1.5°C | 219 | Global TPES | 1.5°C | 998 | 22% |
| | TIAM - 2°C | 225 | | 2°C | 1031 | 22% |
| | WITCH | 243 | | | | 25% |
| **Electricity** | TIAM - 1.5°C | 35 | Global Electricity Production | 1.5°C | 380 | 9% |
| | TIAM - 2°C | 40 | | 2°C | 364 | 11% |
| | WITCH | 54 | | | | 15% |

**Material Use, Sorbent Production:**

| Sorbent Adopted | Assumptions | | Impact at 30 $GtCO_2$ captured | |
|---|---|---|---|---|
| Hydroxide solutions DAC1 | Make-up rate | 0.17-0.29 $t_{NaOH}/t_{CO_2}$[18] | $Gt_{NaOH}$: 5.0-8.55 | $Gt_{Cl_2}$: 4.4-7.6 |
| | Energy for production (13.3 $GJ/ton_{NaOH}$[44]) | 2.2-3.8 $GJ/t_{CO_2}$ | $EJ_{elec}$: 66-114 | |
| Amine sorbents DAC2 | Make-up rate (assumed) | 0.17-0.29 $t_{NaOH}/t_{CO_2}$[18] | $Gt_{ammonia}$: 15-26 | $Gt_{ethyl.oxide}$: 3-5 |
| | Amine syntesis (MEA) | 3.2 $t_{ammonia}/t_{MEA}$[47] | | |
| | | 0.64 $t_{ethyl.oxide}/t_{MEA}$[47] | | |

**Supplementary Table 4. Cost and Energy Estimates for different DACCS Technologies.**
Note that values between brackets are not taken directly from the literature, but a *Capital Recovery Factor (CRF)* has been used to convert from absolute investment cost (*M*$ for a reference plant size) to the annualized value in terms of $/t$CO_2$. *CRF* equal to 0.107 for *DAC1* and 0.119 for *DAC2*, considering a technology-specific discount of 10%, and a plant lifetime of 20 and 15 years respectively.

| Technology | | | CAPEX [M$] | CAPEX [$/t$_{CO_2}$] | OPEX [$/t$_{CO_2}$] | Total [$/t$_{CO_2}$] |
|---|---|---|---|---|---|---|
| | High | [19] | (2060) | 220 | 76 | 300 |
| DAC1 | Low | [11] | 1146 | (140) | 42 | 180 |
| (CRF=0.107) | Floor cost | [11] | 700 | (75) | 27 | 100 |
| | High | [6] | (750) | 90 | 260 | 350 |
| DAC2/21 | Low | [38] | (430) | 50 | 150 | 200 |
| (CRF=0.119) | Floor cost | [69] | (110) | 13 | 37 | 50 |

| Technology | | | Electricity [GJ/t$_{CO_2}$] | Heat [GJ/t$_{CO_2}$] |
|---|---|---|---|---|
| DAC1 | High | [6] | 1.8 | 8.1 |
| | Low | [11] | 1.3 | 5.3 |
| DAC2 | High | [22] | 1.1 | 7.2 |
| | Low | [68] | 0.6 | 4.4 |

**Supplementary Table 5. Waste heat recovery for different industrial sectors.**
Recovery Factor *RF* defined (left) and categories of industrial processes where the waste heat commodity was added (right). For *Other Industries* sector, the recovery factor has been defined as the average of other sectors' values.

| Sector | Recovery Factor Min | Recovery Factor Max |
|---|---|---|
| Iron and Steel | 35% | 32% |
| Pulp and Paper | 30% | |
| Chemicals | 20% | |
| Cement and Glass | 40% | 30% |
| Non-ferrous | 35% | 32% |
| Other Industries | 30% | |

- Gas-fired processes for production of process heat and steam across all different industrial sectors listed in left table. Other fuels than natural gas have been considered only if coupled with a carbon capture unit (see next points)
- CCS processes, burning both natural gas and coal in all sectors listed
- For *Other Industries* sector, processes burning different fuels than natural gas have been considered (e.g. biomass, coal and oil), given that they have still a significant capacity installed up to the end of the century according to the model.

**Supplementary Table 6. Land and Water Use for different NET technologies, according to Smith[5]**

| | **Land Use** $[m^2/t_{CO_2}/yr]$ | | **Water Use** $[t_{H_2O}/t_{CO_2}]$ | |
|---|---|---|---|---|
| | Low | High | Low | High |
| BECCS | 273 | 1636 | 545 | 682 |
| DACCS | 0.1 | 1.50 | 5 | 20 |
| Afforestation | 273 | 1636 | 545 | 682 |

## Supplementary Notes

### Supplementary Note 1. Model Descriptions

*TIAM-Grantham.* The global TIMES Integrated Assessment Model (TIAM) mantained at Grantham Insitute - Imperial College London is a multi-region, least-cost optimization model, minimizing the total net-present value of the global energy system (using by default a 5% time discount rate) to meet future energy service demands up to 2100. Technology-rich models like TIAM-Grantham are particularly of interest to explore the least-cost evolution pathways of the energy system required to meet prescribed climate targets. TIMES is an acronym for The Integrated MARKAL-EFOM System, a model generator for local, national or multi-regional energy systems, developed as a successor of the MARKAL[1] and EFOM[2] bottom-up energy models, and incorporating the main features of these ancestors. This model aims to supply energy services at minimum global cost, when all energy markets are in equilibrium. It is a linear programming bottom-up energy model, that can be coupled with a climate module: demands for different energy services represent the main exogenous driver to simulate the development of global energy economy over time, from resource extraction to the consumption of final useful energy. The region and sector-specific demands for end-use energy or industrial products are driven by socio-economic parameters, such as GDP, population and number of households. Demands for energy services can be elastic to their own prices, thus capturing main feedback from the economy to the energy system[3]. TIAM-Grantham is based on the ETSAP-TIAM framework, which is the global multi-regional incarnation of the TIMES model generator, with a number of sets and processes already defined and built in it, based on the International Energy Agency (IEA) databases and incorporated in TIMES structure within the Energy Technology Systems Analysis Program (ETSAP). Being a technology-rich model, technologies can be modeled purely via data input specification, without having to modify the TIMES source code equations. This makes the model data driven. The economy representation in TIAM-Grantham assumes partial equilibrium on energy markets, with three fundamental properties: linearity, maximization of consumer-producer surplus, and competitiveness of energy markets with a perfect foresight. These properties in turn result in two additional features: marginal cost pricing (at equilibrium, total surplus is maximized), and the profit maximization property. The perfect foresight assumption may be relaxed by assuming that some parameters are uncertain and or running the model in myopic overlapping periods. This assumption is at the basis of the Stochastic Programming option, that has not been used for this work. The objective function is represented by the total discounted aggregate energy system costs summed over all time periods and across all regions. The main cost components included in the objective function are the investment costs, operation and maintenance costs for energy conversion technologies and emission reduction measures, as well as decommissioning expenditures. In TIMES, economic equilibrium conditions determine what technologies are competitive, marginal or uncompetitive in each market, therefore it is a decision made mainly on a system-wide cost-competitiveness basis. TIAM-Grantham include 15 different regions, linked by energy and material trading variables, as well as emission permit trading: trades transform regional modules into a single multi-regional energy model. TIAM-Grantham includes a broad technology database covering many fuel transformation and energy supply pathways and representing technologies based on fossil, nuclear and renewable energy resources. Both currently applied technologies and future advanced technologies, such as hydrogen technologies and options for carbon dioxide capture and storage (CCS) in power plants and industrial applications, are available in the model's technology portfolio. With regard to climate change mitigation measures, the model covers both $CO_2$ emissions, related to energy consumption, and $CH_4$, from energy consumption as well as from some non-energy sectors, such as landfills, manure, wastewater or biomass burning. In addition, $N_2O$ from energy consumption as well as from acid industries can be modeled. TIAM-Grantham is able to simulate different types of emission abatement measure, such as energy substitution within the available portfolio, improved efficiency of installed device, sequestration ($CO_2$ capture and underground storage, biological carbon sequestration), regulations and taxes, as well as a cap-and-trade system. Indeed, endogenous trade of all emissions is available, so to model permit trading.

*WITCH.* The WITCH (World Induced Technical Change Hybrid) model is a global integrated assessment model maintained at RFF-CMCC European Institute on Economics and the Environment. Being a hybrid model, it combines a top-down inter-temporal optimal growth model, a regional game-theoretic setup, a bottom-up representation of the energy sector, and an endogenous treatment of technological innovation. Technological change is accounted for both via learning curves influencing the investment cost of new vintages of capital and via R&D investments. The economy and energy models are hard-linked, so that energy investments and resources are chosen optimally considering the trend of macroeconomic variables[4,5]. Differently, land use management and future climate are soft linked, so they are available through GLOBIOM (Global Biosphere Management Model)[6] and MAGICC (Model for Assessment of Greenhouse Gas Induced Climate Change)[7], which are respectively a land use and forestry model and a climate model. The climate model is used to compute climate variables from GHG emission levels and to account for climate feedback on the economy to determine the optimal adaptation strategy, accounting for both proactive and reactive adaptation expenditures. The objective of WITCH is to maximize the discounted utility for every region. The utility function has as output a final good that derives from capital and labour, according to a Cobb-Douglas productivity law, combined with energy services through a CES (Constant Elasticity of Substitution). The

regional utility function is also influenced by other parameters such as population, time preference discount factor and final good consumption. The regional and intertemporal dimensions of the model make it possible to differentiate and assess the optimal response to several climate and energy policies across regions and over time. The non-cooperative nature of international relationships is explicitly accounted for via an iterative algorithm which yields the open-loop Nash equilibrium between the simultaneous activity of a set of representative regions. The model has a time horizon that goes from 2005 to 2150 with a time step of 5 years. In its default configuration, the world is divided into 13 regions, each including countries with similar economic situations, energy systems, climate policies and political strategies. If regions are facing a global policy target, they can either behave independently or form coalitions: in the second case, coalitions of regions optimize their total welfare as a whole. In this study we assumed each regions to behave independently. WITCH includes several technologies in order to describe the energy sector. Every technology can participate to the energy mix production thanks to a CES structure, so there is the possibility to switch from a technology to the other according to their elasticity of substitution. Electricity can be generated by traditional fossil fuel plants and low-carbon sources. It is present also a system integration module that guarantees flexibility and capacity constraints on variable renewables energies (VRE). R&D investments are directed towards either energy efficiency improvements or development of carbon-free breakthrough technologies. Such innovation cumulates over time and spills across countries in the form of knowledge stocks and flows.

## Supplementary Note 2. Time Discount Rate in TIAM-Grantham and WITCH

Due to the dissimilar model characteristics previously discussed, time and inter-generational preference are accounted differently between both models.

In TIAM-Grantham, the objective function is the minization of the total system cost, with future cost elements appropriately discounted to a selected reference year. The general discount rate applied is defined exogenously, and by default it is set as equal to 5%, while in the LowDiscount scenario it is reduced to 0.1%. In addition, there are also technology-specific hurdle rates (ranging from 2 to 18%), which have not been modified here.

In WITCH, due to the more refined macro-economic description, a standard Ramsey model is used to define endogenously the discount rate $\rho$ as:

$$\rho = \delta + \eta \cdot g$$

where $\delta$ is the pure rate of time preference (i.e. the only value considered in TIAM-Grantham), $\eta$ is the elasticity of the marginal utility of consumption and $g$ is the average growth rate of consumption, that in the long-term tends to 1.6% in SSP2 scenarios[1]. Default values are $\delta$=1.0% and $\eta$=1.5, leading to an overall discount rate $\rho$ around 3.5%. Differently, in LowDiscount runs, we set $\delta$ = 0 and $\eta$=0.1, thus obtaining an overall discounting around 0.15%, consistently to what has been implemented in TIAM-Grantham.

## Supplementary Note 3. Storage Capacity in TIAM-Grantham and WITCH

When running sensitivity analysis on storage availability, we need to account for the different database implemented in our models. It should be noted that in both cases the following storage options are included: saline aquifers (on/off-shore), depleted oil and gas fields (on/off-shore), enhanced oil recovery (on/off-shore), enhanced coal bed methane.

In particular, TIAM-Grantham high, best and low estimates from Hendricks[8] are used to define storage potential scenarios, with a global capacity around 5,000, 1,500 and 500 Gton$CO_2$ respectively, then allocated to each sequestration option and each region. The baseline case assumes a global capacity around 9000 Gt$CO_2$, while the best estimate from Hendriks has been used when running the LowStorage sensitivity, as the low case results too stringent for the model to find a feasible solution. Differently, in WITCH regional storage capacity limits are defined combining different sources[8–12], so to gather updated information about the potential for different storage categories. The resulting global capacity is around 20,600 (high), 11,000 (best) and 4,900 (low) Gton$CO_2$: the best case represents the default value implemented, while the low scenario has been adopted for storage sensitivity.

## Supplementary Note 4. CCS and BECCS Cost Assumptions in TIAM-Grantham and WITCH

As we have already discussed, TIAM-Grantham results to be highly sensitive to costs when choosing the technology portfolio to be deployed, therefore it has been decided to update costs for BECCS and CCS plants and to align them to the ones used in WITCH, properly accounting for the different technologies represented within the two models. In particular, gas-based plants with oxy-fuel are implemented only in TIAM-Grantham, therefore their cost is estimated considering the same percentage difference between price of post-combustion and oxy-fuel capture processes for coal plants in WITCH. At the same time, bioenergy power plants in WITCH are represented by one single technology, while in TIAM-Grantham we can find further differentiation between plants based on biomass gasification and direct combustion, therefore the reference costs for these two

[1]Note that GDP and population growth are calibrated against SSP2 as a baseline in WITCH

technological options have been taken from the IEAGHG Report[13].Note that in both model initial and floor costs have been identified, so to put a lower bound to future cost reduction due to exogenous (in TIAM-Grantham) or endogenous technical learning (in WITCH). All these values are summarized in Supplementary Table 6.

### Supplementary Note 5. Impact of DACCS in transport and industry sectors.

As it has been already highlighted, one of the main advantages of deploying DACCS plants is the possibility to capture the $CO_2$ produced by distributed sources such as residential heating/cooling, transportation (aviation and road transport) and energy-intensive industries, such as steel and cement manufacture. These account for almost 50% of the total[?]. Indeed, collecting carbon dioxide from small burning units at the source often results difficult and not economical with large CCS facilities. DACCS is not the only way to abate these emission sources, but the cost of the alternatives, such as electrification or increased the use of biomass, are expected to be considerably higher than the current estimates for Direct Air Capture facilities[?]. Indeed, looking at Supplementary Figure 2 it can be noted that higher residual emissions are actually allowed from sectors such as transport and industry when a full portfolio of NET technologies is available, rather than when relying only on BECCS and afforestation. On the one hand, this limit a rapid and massive expansion of intermittent renewable sources, with an impact on grid security, storage and additional cost for the entire system (see Figure **??**). On the other one, it is interesting to consider how this is reflected in terms of Total Primary Energy Supply (TPES). From Supplementary Figure 2, it can be noted that the availability of Direct Air Capture allows a higher share of fossil fuel in the mix along the century, in a similar way as in the electricity production mix shown in Figure **??**. The larger amount of oil, coal and gas in the TPES reflects the use of fossil resources in other sectors than the electricity generation. In particular, these are employed as fuels for industrial processes and transport activities. When DACCS is not available within the mitigation portfolio (NoDAC and NoNET scenarios), these emission sources are mitigated through a massive electrification of transport and building sectors (reflected in the higher global electricity demand in Figure **??**) and an increased use of advanced biofuels (reflected in the larger share of biomass in the primary energy supply in Supplementary Figure 2). Conversely, a full NET portfolio (DAC scenario) allows a less drastic transition in these sectors, with the share of oil and coal being not too far from the baseline (BAU) scenario. The total amount of primary energy needed throughout the century increases when DACCS is available, due to the large energy requirements to operate these plants. Overall, this increase appears limited, being up to 15% higher in DAC scenarios with respect to NoDAC ones.

### Supplementary Note 6. Results from Sensitivity Analysis

In base scenarios, among the different DACCS options considered in our study, the one based on solid amine sorbents (DAC2) is generally deployed earlier in time, while DAC1 becomes competitive later in the century, when larger amount of natural gas is available at cheaper prices to fuel it, as the role of fossil-based electricity generation is strongly reduced. The relative share of these technologies changes a lot across sensitivity scenarios, as it can be seen from Supplementary Figure 2b.

According to both models, the influence of **energy and cost parameters** is limited in determining the overall DACCS deployment. Indeed, the models tend to install this technology as a backstop solution to meet the climate target imposed, regardless of the costs associated. The only case where we can find a significant change in DACCS capacity is in TIAM-Grantham, with a 2°C target: lower energy consumption brings 125 $GtCO_2$ more captured throughout the century. Differently, with a more stringent target the models already deploy this technology at the highest rate allowed. Therefore, the impact is not visible in terms of cumulative sequestration but it affects the share of DACCS technologies: in LowEnergy scenarios, DAC1 is favored over amine-based plants, given that energy expenditure represents a significant fraction of the total operational cost for this option (see Supplementary Figure 2b).

Differently, **growth constraints** are the most influencing and critical parameters in our modeling exercise. The level of DACCS capacity deployed depends mainly by the overall cap lmiit applied exogenously: while BECCS is limited to a capture rate around 10 $GtCO_2$/yr by bioenergy availability (around 200 EJ/yr in both models), there are no external limiting factors that can be applied to DACCS a priori, so that the choice of the maximum capacity limit is highly arbitrary. As we explain in the main text, we investigate the impact of different capacity limits on DACCS, namely 30 and 3 $GtCO_2$/yr for the high and the low case. These values roughly correspond to scaling the current market for hydroxide production (i.e. the sorbents adopted in DAC1 plants) by a factor of 100 and 10 respectively. Indeed, sorbent production and availability may be a critical factor when large scale deployment is reached. When limiting DACCS capture capacity to 3 Gt/yr, the impact on net emission pathways is very marked, as it is required a strong reduction of residual emissions in the mid-term (Figure **??**). Indeed, in these scenarios DACCS cumulative sequestration is reduced by between 550 and 770 $GtCO_2$, while BECCS plants are capturing about 20 to 50% more $CO_2$ along the century, with a significant impact also on the energy sector (Supplementary Figure 3). Indeed, the drastic transition towards low-carbon electricity generation is very similar to the one observed in NoDAC scenarios, with a major role played by natural gas plants (with CCS) in the short term, and a larger deployment of renewables by mid-century.

Increasing the annual growth rate, a Gt-scale deployment is reached earlier in time, capturing up to 480 $GtCO_2$ more throughout the century with a 1.5°C target. On the other hand, limiting the pace of scale-up to only 15% per year reduces the cumulative capture of more than 400 $GtCO_2$ across all cases, affecting mainly DAC2 which is the technology generally deployed earlier in time (see Supplementary Figure 2b).

Due to the perfect foresight assumed by our models, the availability of NETs deeply influences the timing of mitigation. The parameter defining inter-generational preferences in IAMs is the time discount rate. As we have highlighted before, a lower **discount rate** leads to earlier decarbonization in both models, limiting the need for carbon dioxide removal at the end of the century (see Supplementary Figure 4a). This is more visible with a 2°C target, rather than a 1.5°C one (supplementary Figure 4a), arguing that all the alternatives to remove $CO_2$ from the atmosphere needs to be deployed at their maximum potential to meet ambitious climate goals. Considering the energy sector, this implies a reduced role for coal in the electricity mix, with its complete phase-out already in 2030 with a 1.5°C target, being replaced by gas-based generation and renewables in the long run (Supplementary Figure 6). The lower the discount rate, the more DAC2 plants are negatively affected from 2070 onward. Nevertheless, even when drastic emission reduction is applied in the earlier decades, DACCS will still play a role in the second half of the century to reduce the impact of mitigation on energy and industrial sectors. This suggests that DACCS will be needed more if mitigation is delayed, while more drastic short-term mitigation efforts could reduce a massive deployment.

We run a sensitivity analysis on **storage capacity** to understand whether it represents a bottleneck for the diffusion of DACCS and its competition with other sequestration options. Indeed, when the availability of sequestration sites for $CO_2$ is limited, priority is given to DACCS over the other sequestration options, with the cumulative capacity by BECCS plants being 30 to 45% lower than in the base case. The deployment of BECCS and traditional CCS is strongly reduced around mid-century, with about 3 $GtCO_2$/yr less captured by bioenergy plants and traditional CCS being limited to sequester 2-3 $GtCO_2$/yr. In TIAM-Grantham this results is lower emissions between 2020 and 2050 (Supplementary Figure 5). There is a significant impact also on the carbon price, being about twice as large as in the base case, and on the energy sector (see Supplementary Figure 6). Indeed, when storage capacity is limited the role of coal with CCS in the electricity mix is markedly diminished, while gas-based CCS plants are preferred and more renewables are installed. This is more evident in TIAM-Grantham rather than in WITCH, as in this model LowStorage estimates are more stringent (as discussed in Supplementary Note 3). It is interesting to note that this is the only case where also DAC21 technology is being installed in TIAM-Grantham (see Supplementary Figure 2b), that represents amine-based plants fuelled by heat made on purpose instead of waste heat from industrial and renewables plants. This can be related to the reduced BECCS deployment, so that biomass supply is available at cheaper price to produce electricity and heat for DAC21 with a low-carbon technology, Indeed, in TIAM-Grantham most of the low-temperature heat is being produced by biomass-based CHP plants (i.e. producing electricity and heat at the same time): in all other scenarios, biomass supply is used mainly to fuel BECCS as it is not convenient, nor efficient, for the model to use this limited resource to produce the heat to capture $CO_2$ with amine-based plants. Differently, with a limited storage availability and a reduced role for BECCS, a large amount of bioenergy supply is available at cheaper price to produce the heat to fuel DAC21.

## Supplementary References

## References


1. Fishbone, L.G. Abilock, H. Markal, a linear-programming model for energy systems analysis: Technical description of the bnl version. *International Journal of Energy Research*, 1981.

2. Vander Voort, E.The EFOM 12C energy supply model within the EC modelling system. *Omega*, 1982.

3. Loulou, R. Labriet, M. ETSAP-TIAM: The TIMES integrated assessment model Part I: Model structure. *Computational Management Science*, 2008

4. Bosetti, V., De Cian, E., Sgobbi, A. & Tavoni, M. The 2008 Witch Model: New Model Features and Baseline (2009). DOI: 10.2139/ssrn.1512475.

5. Bosetti, V., Carraro, C., Galeotti, M., Massetti, E. & Tavoni, M. WITCH: A World Induced Technical Change Hybrid model. *Energy J.* DOI: 10.2139/ssrn.948382 (2006).

6. Havlík, P. et al. Global land-use implications of first and second generation biofuel targets. *Energy Policy* **39,** 5690–5702 (2011).

7. Meinshausen, M. Raper, S.C.B. Wigley, T.M.L. Emulating coupled atmosphere-ocean and carbon cycle models with a simpler model, MAGICC6 – Part 1: Model description and calibration. *Athmos. Chem. Phys.*, **11,** 1417-1456 (2011)

**8.** Hendriks, C. Graus, W. Global Carbon Dioxide Potential and Costs, Ecofys, TNO, Tech. Rep. (2004) [Online]. Available: www.ecofys.nl

**9.** IEAGHG. Potential for biomass and carbon dioxide capture and storage. IEA Greenhouse Gas R&D Programme. (2011)

**10.** IEA. CO2 Emissions from Fuel Combustibles. International Energy Agency. *OECD-IEA Publ.* DOI: 10.1787/co2-table-2011-1-en (2015)

**11.** Godec, M., Kuuskraa, V., Van Leeuwen, T., Melzer, L. S. & Wildgust, N. CO2storage in depleted oil fields: The worldwide potential for carbon dioxide enhanced oil recovery. *Energy Procedia* DOI: 10.1016/j.egypro.2011.02.102 (2006).

**12.** Dooley, J.J., Estimating the supply and demand for deep geologic CO2storage capacity over the course of the 21stcentury: A meta-analysis of the literature. *Energy Procedia* DOI: 10.1016/j.egypro.2013.06.429 (2013).

**13.** Koornneef, J., et al. Global potential for biomass and carbon dioxide capture, transport and storage up to 2050. *Int. J. Greenh. Gas Control.* DOI: 10.1016/j.ijggc.2012.07.027 (2012)

78**.** International Energy Agency (IEA). *Snapshot of global PV 1992–2014.* Photovoltaic power systems programme. (2015)
